# Phenolic Profile and Biological Activities of the Pepino (*Solanum muricatum*) Fruit and Its Wild Relative *S. caripense*

**DOI:** 10.3390/ijms17030394

**Published:** 2016-03-16

**Authors:** Francisco J. Herraiz, Débora Villaño, Mariola Plazas, Santiago Vilanova, Federico Ferreres, Jaime Prohens, Diego A. Moreno

**Affiliations:** 1 Instituto de Conservación y Mejora de la Agrodiversidad Valenciana, Universitat Politècnica de València, Camino de Vera 14, 46022 Valencia, Spain; fraherga@upvnet.upv.es (F.J.H.); maplaav@btc.upv.es (M.P.); sanvina@upvnet.upv.es (S.V.); 2 Centro de Edafología y Biología Aplicada del Segura–Consejo Superior de Investigaciones Científicas, Food Science and Technology Department, Research Group on Quality, Safety and Bioactivity of Plant Foods. Campus Universitario de Espinardo–25, Espinardo, 30100 Murcia, Spain; dvillano@ucam.edu (D.V.); federico@cebas.csic.es (F.F.); dmoreno@cebas.csic.es (D.A.M.)

**Keywords:** antioxidants, biological activity, HPLC-DAD-MS^n^/ESI, macrophages

## Abstract

The pepino (*Solanum muricatum*) is an edible and juicy fruit native to the Andean region which is becoming increasingly important. However, little information is available on its phenolic composition and bioactive properties. Four pepino varieties (37-A, El Camino, Puzol, and Valencia) and one accession (E-7) of its close wild relative *S. caripense* were characterized by HPLC-DAD-MS^n^/ESI. Twenty-four hydroxycinnamic acid derivatives were detected (5 to 16 compounds per variety or accession), with differences of more than two-fold for their total content among the materials studied. The major phenolics in the pepino varieties were chlorogenic acids and derivatives, while in *S. caripense* a caffeoyl-synapoyl-quinic acid was the major compound. The *in vitro* antioxidant capacity (DPPH (2,2-diphenyl-1-picrylhydrazyl hydrate), ORAC (oxygen radical absorbance capacity), and TRC (total reducing capacity) tests) was higher in *S. caripense*. Pepino and *S. caripense* extracts were not toxic for RAW 264.7 macrophage cells, and the raw extracts inhibited NO production of the lipopolysaccharide (LPS)-stimulated macrophages by 36% (El Camino) to 67% (37-A). No single variety ranked high simultaneously for hydroxycinnamic acids content, antioxidant activity and biological activity. We suggest the screening of large collections of germplasm or the use of complementary crosses between Puzol (high for hydroxycinnamic acids and biological activity) and *S. caripense* E-7 (high for antioxidant activity) to select and breed pepino varieties with enhanced properties.

## 1. Introduction

The pepino (*Solanum muricatum* Aiton), also known as pepino dulce, is an herbaceous Andean crop cultivated for its edible, mildly sweet, and juicy fruits [1]. The pepino’s fruits may be variable in fruit size, shape, and color [2], but they generally weigh between 80 and 250 g, are round to elongated in shape, and have a yellow skin with purple (when immature or ripe) or brown (when fully ripe) longitudinal stripes that cover a variable part of the fruit surface [3,4,5]. The pepino fruits are considered very refreshing, as they have a high moisture content (typically above 90%), and are very aromatic [5]. Pepino cultivation was important during pre-Columbian times, but since the decline of the Inca Empire it increasingly became a neglected crop [6]. However, during the past few decades there has been renewed interest in pepino cultivation both in the Andean region and in several other countries, as the pepino is considered a crop with potential for diversification of horticultural production [3,5,7].

Apart from its attractive morphological features, the pepino fruit has been attributed antioxidant, antidiabetic, anti-inflammatory, and antitumoral activities [8,9,10]. An important feature for the enhancement and increase of the demand of exotic fruit crops like the pepino is having knowledge on the composition of biologically active constituents and the discovery of properties that may be of interest for human health [11], which may stimulate demand. It is known that pepino fruits contain significant amounts of vitamin C, as well as carotenoids, which give the yellow color to the flesh [8,12]. However, for phenolic compounds, which make a major contribution to the bioactive properties of other *Solanum* fruits like the tomato (*S. lycopersicum* L.), tree tomato (*S. betaceum* L.), or common (*S. melongena* L.), scarlet (*S. aethiopicum* L.), and gboma (*S. macrocarpon* L.) eggplants [13,14,15,16], there is little information on the pepino [8,12,17]. In this respect, it has been found that the phenolics content of the pepino fruit is much higher than that of vitamin C [8,12], indicating that they may have an important role in the pepino’s bioactive properties. Regarding the phenolics profile, Hsu *et al.* [8] using HPLC separation detected five phenolic acids and four flavonoids, while Wu *et al.* [17] used LC-TOF-MS methods to study the phenolic profiles of several *Solanum* species, including the pepino, and were able to detect eight hydroxycinnamic acid derivatives and one flavonoid in the pepino fruit. All these studies used only one variety and therefore little information exists on the diversity of pepino phenolics.

Up to now, most of the breeding efforts in pepino have been devoted to improving yield, resistance to diseases, and fruit flavor and aroma [3,5]. Also, some works reveal that there is an important diversity in vitamin C content [18]. However, up to now no comprehensive studies exist on the diversity of phenolics compounds and their concentration in the pepino fruit. In this respect, breeding for other fruit quality properties, like antioxidant activity and biological activity, as well as its relationship with the phenolics content, would be of great relevance for the enhancement of this crop. However, again, no information is available on the diversity for these traits, as all studies are based on a single variety [8,9,19].

In this work, we determine the phenolic profile and content of pepino fruits using HPLC-DAD-MS^n^/ESI, and study the antioxidant and biological (anti-inflammatory) activities of a diverse set of pepino varieties. We also include one *S. caripense* accession, which is a close wild relative of the pepino [1,20] that has been used for pepino breeding [4,5]. The information obtained will provide relevant information on the phenolic profile and composition of pepino fruits and will be of great interest for the selection, breeding, and enhancement of this crop.

## 2. Results

### 2.1. Phenolic Composition

**Figure 1:**
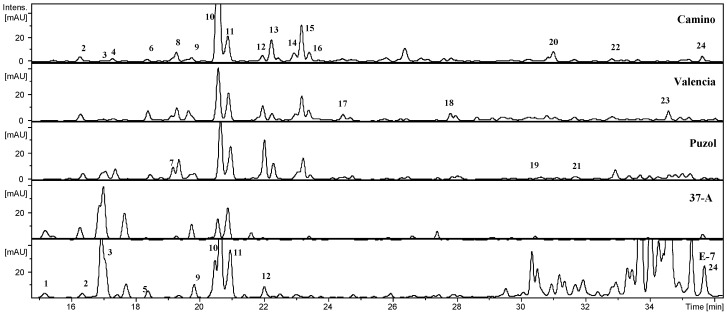
Chromatogram obtained from reversed-phase LC-MS/MS analysis of pepino varieties. Numbers in bold correspond to the peaks identified and described in Table 2 and Table 3.

Based on retention times, UV spectra, [M − H]^−^, and mass fragmentation and comparison with available data in the literature [21,22,23,24], a total of 24 phenolic compounds were identified in the five accessions of *S. muricatum* and *S. caripense* (Table 1). All the compounds detected corresponded to hydroxycinnamic acid derivatives (Table 1). The chromatogram at 320 nm shows a high abundance in most varieties of peak **10**, corresponding to 5-caffeoyl-quinic acid (chlorogenic acid) (Figure 1). Peak **11** shows, like peak **10**, a deprotonated molecular ion *m*/*z* 353, but the MS fragmentation pattern reveals that it corresponds to 4-caffeoyl-quinic acid. Peaks **2** and **14** were identified also as other caffeoyl-quinic acids, by means of MS^2^ of their deprotonated molecular ion (*m*/*z* 353), having a base peak at *m*/*z* 191. The compound **2** also gave a relative intense ion at *m*/*z* 179; however, in the compound **14** this ion is undetectable. According to Clifford *et al.* [21], they can be labeled as the 3-caffeoyl-quinic acid and 5-caffeoylquinic acid isomers, respectively. Di-caffeoyl-quinic acids were detected at retention times of 15 min (peak **1**), 18 min (peak **5**), 20 min (peak **19**), and 33 min (peak **22**), with deprotonated molecular ion *m*/*z* 515 [24]. Peaks **3**, **4**, **6**, and **9** were identified as caffeoyl-hexosides (*m*/*z* 341), with similar fragmentation patterns. Other caffeoyl-hexosides identified corresponded to peak **8** as caffeoyl-di-hexoside (*m*/*z* 503), and peaks **18** (*m*/*z* 441) caffeoyl-hexoside derivatives because their MS fragmentation gave a *m*/*z* 341 ion (deprotonated caffeoyl-hexosides). The compound **23** (*m*/*z* 433) should be a caffeoyl derivative because in its MS fragmentation we could find the ions at *m*/*z* 179 (deprotonated caffeic acid) and 135 (179-44). Feruloyl-hexosides (compounds **7**, **13**, and **17**, *m*/*z* 355), feruloyl-di-hexosides (**15** and **21**, *m*/*z* 517) and *p*-coumaroyl-di-hexoside (peak **12**, *m/z* 487) were also identified. We also detected sinapoyl derivatives, with *m/z* 547 (peak **16**, sinapoyl-di-hexoside), and peak **20** (*m/z* 577), its MS fragmentation gave the *m/z* 415 ([(M − H) − 162]^−^, loss of the caffeoyl-radical), 353 (deprotonated caffeoyl quinic acid, [(M − H) − 224]^−^) as well as the 224 (neutral sinapic acid) and 191 (deprotonated quinic acid); therefore, the compound is a sinapoyl-quinic acid derivative, and in the *m/z* 559 (peak **24**) with a MS fragmentation including a loss of 162 amu (caffeoyl-radical) to give the ion at *m/z* 397, the deprotonated sinapic acid (*m/z* 223) and the ion at *m/z* 173 ([quinic acid-H-18]^−^) is also observed, and therefore is a caffeoyl-sinapoyl-quinic acid.

**Figure 2:**
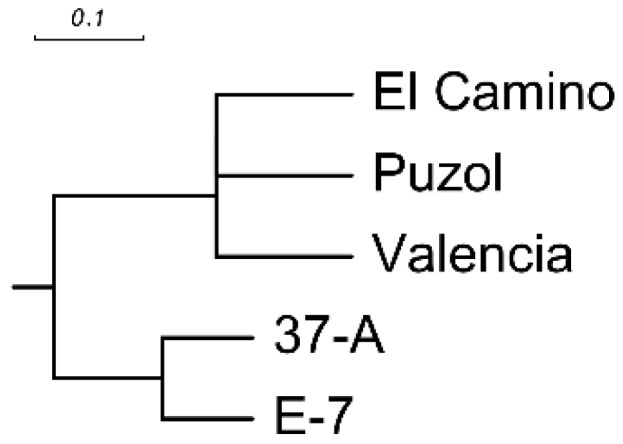
Euclidean distance-based UPGMA phenogram of four pepino varieties (37-A, El Camino, Puzol, and Valencia) and one *S. caripense* accession (E-7) according to the absence/presence of 24 phenolic compounds detected by HPLC-DAD-MS^n^/ESI.

The number of phenolic compounds detected per variety ranged between five for pepino accession 37-A, and 16 for pepino accessions El Camino, Puzol, and Valencia, while *S. caripense* E-7 presented nine identifiable compounds (Table 1, Figure 1). Out of the 24 identified compounds, 3-caffeoyl-quinic acid, caffeoyl-hexose IV, 5-caffeoylquinic acid and 4-caffeoyl-quinic acid were present in all accessions. On the other hand, some compounds were specific of accession. In this respect, three compounds (peaks **14**, **16**, and **20**) were specific to El Camino, two compounds (peaks **17** and **18**) to Valencia, two (peaks **1** and **4**) to *S. caripense* E-7, and one (peak **19**) to Puzol, while no compounds were specific to 37-A (Table 1). The dendrogam obtained based on the presence/absence of the 24 phenolic compounds reveals two major groups: one constituted by the wild *S. caripense* E-7 and the primitive pepino variety 37-A, while the other includes the three modern pepino cultivars (El Camino, Puzol, and Valencia) (Figure 2).

Thirteen of the 24 compounds were present in sufficient quantities to be detected in at least one of the accessions (Table 2). In some cases, the concentrations were lower than the limit of quantification and therefore could not be quantified. Total contents of hydroxycinnamic acids ranged between 1.11 mg/g (37-A) to 2.35 mg/g (Valencia). All the accessions were characterized for presenting a predominant compound, which represented between 59% and 82% of the total content in hydroxycinnamic acids. Three different preeminent compounds were detected depending on the accession. In pepino varieties, two dicaffeoylquinic acids, namely the isomers 3-caffeoyl-quinic acid (variety 37-A) and 5-caffeoyl-quinic acid (varieties El Camino, Puzol, and Valencia) were the major compounds. For *S. caripense* E-7 the major hydroxycinnamic acid derivative was a caffeoyl-synapoyl-quinic acid (Table 2), which was also present in quantifiable quantities in the pepino varieties and was the second most abundant compound in pepino variety 37-A. For the rest of the pepino varieties, the second major compound was feruloyl-dihexose (El Camino), feruloyl-hexose (Puzol), and *p*-coumaroyl-di-hexose (Valencia). These three compounds were present in these three varieties at significant levels, with the exception of feruloyl-hexose in Valencia.

### 2.2. Antioxidant Activity

Significant differences have been found among the five accessions studied, with a range of variation of 3.3-, 1.6-, and 1.9-fold for the ORAC (oxygen radical absorbance capacity), DPPH (2,2-diphenyl-1-picrylhydrazyl hydrate) free radical scavenging capacity, and TRC (total reducing capacity) assays, respectively (Table 3). ORAC values have been always higher (on average 4.7-fold) than those of DPPH, despite being measured in the same units (μmol Trolox/g). The highest values for the three methods have been obtained for *S. caripense* accession E-7. For the ORAC method the values obtained by E-7 have been more than two-fold greater than the pepino accession with highest ORAC values (37-A), which presented values 1.6-fold higher than Valencia, which was the pepino variety with the lowest ORAC values (Table 3). For DPPH free radical scavenging capacity, the differences between *S. caripense* E-7 and the pepino varieties have been much lower, and in fact pepino variety Puzol presented values similar to those of E-7 (Table 3). The Puzol variety had DPPH free radical scavenging capacity values 1.6-fold higher than those of El Camino, which was the variety with the lowest values for this antioxidant parameter. Finally, for TRC all the pepino varieties presented significantly lower values than those of *S. caripense* E-7. In this case, the pepino variety with the highest values was 37-A, with values 1.5 fold higher than Puzol, which was the variety with the lowest value for this parameter (Table 3).

### 2.3. Biological Activity

**Figure 3:**
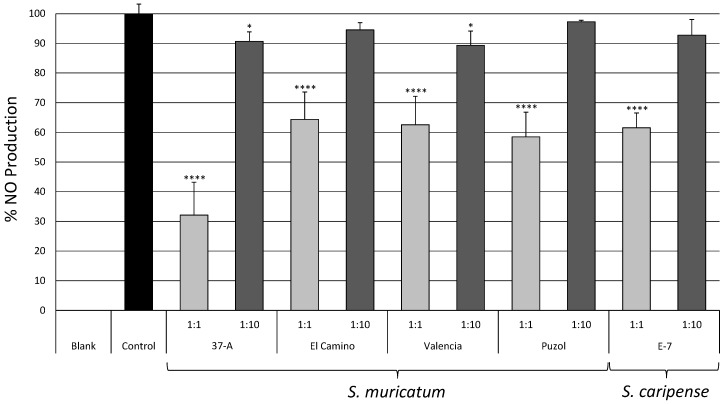
Percentage of nitric oxide (NO) production of lipopolysaccharide (LPS) stimulated RAW 264.7 macrophages incubated in raw (1:1; light grey columns) and diluted (1:10; dark grey columns) methanolic extracts of pepino and *S. caripense* accessions compared to a control with no extract (relative production of the control is assigned a value 100%). Bars represent ± SE (*n* = 5) of the mean. Columns tagged with asterisks indicate that the mean values are significantly different from the control (**** and * indicate significance at *p* values of 0.0001 and 0.05, respectively) according to the Dunnett’s multiple comparison test.

No significant differences were observed over the controls on cell viability of macrophage cells of raw (1:1) and diluted (1:10) extracts of pepino and *S. caripense*, revealing a lack of toxicity on these cells of any of the extracts. Consequently changes induced by these extracts on nitric oxide (NO) production cannot be explained by cell death caused by the extracts. All raw extracts demonstrated a significant inhibition of the NO production by the macrophage cells (Figure 3). The greatest inhibition of NO production was caused by pepino variety 37-A, with 67% of inhibition with respect to the control, while the other varieties had a similar performance, with inhibition values ranging from 36% (El Camino) to 41% (Puzol). The 1:10 dilutions of pepino varieties 37-A and Valencia also presented significant inhibition of the NO production, but the values were much lower (always below 10%) than those of the raw extracts (Figure 3). The 1:10 dilutions for the rest of the pepino varieties (El Camino and Puzol) and *S. caripense* E-7 did not result in a significant inhibition of NO production.

### 2.4. Selection of Varieties for Phenolic Content and Biological Activities

When varieties are ranked for their total content in total hydroxycinnamic acids, ORAC, DPPH and TRC antioxidant activities and inhibition of NO production in stimulated macrophage cells we did not find a single variety ranking high for all traits considered (Table 4). On the other hand, one variety (El Camino) generally was ranked low, with an intermediate rank (3) for hydroxycinnamic acids content and a low rank (4 or 5) for the antioxidant traits and NO production inhibition. Pepino variety Valencia, which ranked first for hydroxycinnamic acids content, also presented intermediate or low ranks for the rest of traits (Table 4). *Solanum caripense* E-7 had the highest ranks for the three antioxidant measures, but presented a low rank for hydroxycinnamic acids content and an intermediate rank for NO production inhibition. Pepino variety 37-A ranked first for NO production inhibition and second for ORAC and TRC antioxidant measures, but presented the lowest rank for hydroxycinnamic acids content and a low rank for DPPH antioxidant activity. Finally, pepino variety Puzol ranked second for hydroxycinnamic acids content, DPPH antioxidant activity and NO production inhibition, with an intermediate rank for ORAC and the lowest rank for TRC (Table 4).

## 3. Discussion

This is the first study in which phenolics profile and composition, antioxidant activity, and biological activity have been studied in several pepino varieties and in its wild relative *S. caripense*. The HPLC-DAD-MS^n^/ESI technique, which is very efficient for detecting and identifying phenolic compounds of plant extracts, has allowed detection of 24 hydroxycinnamic acid derivatives in the pepino and *S. caripense* fruit flesh. The selection of this method is based on previous experiments [24,25], in which we observed that methanol improves the phenolic acid ionization in the LC-MS compared to ethanol, which extracts more sugars from the plant matrix and therefore is less suitable for this type of study. This substantially increases the number of phenolic metabolites detected up to now in the pepino [8,17], with a number of phenolic metabolites similar to those detected in the tomato using the same technique [24], significantly improving the phytochemical characterization of pepino varieties.

Amazingly, all the phenolic compounds detected corresponded to hydroxycinnamic acid derivatives; no flavonoids were identified. This indicates that the pepino flesh is more similar in phenolic composition to the eggplant, whose phenolic fraction is mostly constituted by hydroxycinnamic acid derivatives [26,27], than the tomato, which also has significant quantities of flavonoids [16,24,26,28]. Our results agree with those obtained by Wu *et al.* [17], who found that hydroxycinnamic acid derivatives were the major phenolic compounds of pepino flesh and only detected one flavonoid (isoquercitrin) at very low concentration in the flesh of the pepino. However, Hsu *et al.* [8] reported significant levels of flavonoids such as myricetin, naringenin, quercetin, and rutin in aqueous and ethanolic extracts of pepino. These discrepancies may be caused by differences in the plant material used and/or the extraction and detection methodology [29,30].

The high diversity found among the five accessions used for the profile of phenolic acids corresponds with the high genetic diversity of the cultivated pepino and its close wild relatives [2,7,18,20]. Only five compounds were universal to all the accessions and eight compounds were specific of accession, which indicates that as in other *Solanum* fruit species, like the eggplant [17], fruit phenolic acids profile may be useful for chemotaxonomy and evaluating relationships in the pepino group. In fact, the separation in the UPGMA dendrogram of the three modern pepino varieties in one cluster and the *S. caripense* E-7 accession and the pepino variety 37-A in another cluster is in agreement with a DNA markers study, in which 37-A was intermediate between regular pepino cultivars and wild species [2]. Morphological data also support the idea that 37-A is a primitive variety, which probably presents introgressions from wild species. Amazingly, the wild *S. caripense* E-7 and the pepino cultivar 37-A have fewer phenolic compounds and lower concentration than the modern varieties, which is in contrast to what has been found in the eggplant and tomato, in which the domestication and breeding processes have reduced the phenolics content [31,32,33].

The predominant phenolic compounds of pepino, as it occurs in many vegetables [34], have been the chlorogenic acid isomers caffeoylquinic acid and 3-caffeoyl-quinic acid. However, for *S. caripense* E-7 the major compound has been caffeoyl-sinapoyl-quinic acid, which is characteristic of *Robusta* coffee [35]. This suggests that important biochemical differences must exist in the pathway of synthesis of phenolic acids between the pepino and *S. caripense*.

The three antioxidant measures taken involve hydrogen atom transfer (ORAC) or electron-transfer (DPPH and TRC) reactions [36]. ORAC values of pepino samples have been much higher than those of DPPH, an observation also found in other fruits like in citrus [37]. The antioxidant capacity of pepino varieties depends both on the antioxidant activity of each phenolic compound as well as the concentration present, the possible synergisms, and the method employed. In consequence, the differences observed in the rank order among the three antioxidant tests employed (ORAC, DPPH, and TRC) may be caused by differences in the antioxidants present in the materials studied. The ORAC method employs a more hydrosoluble environment than DPPH, suitable for compounds as the hydroxycinnamic acids of pepino samples. By comparison with other fruits and vegetables, ORAC values are intermediate-high [38,39]. The Folin–Ciocalteu method is commonly used for estimating the total phenolics content of fruits and vegetables, although it really measures the total reducing capacity (TRC) [40]. In our case the antioxidant values measured by the TRC method using the Folin–Ciocalteu reagent revealed that the antioxidant activity of the pepino is comparable to that of the eggplant [41] which has a high antioxidant capacity [42]. These data indicate that the pepino presents high values for antioxidant capacity and may make a significant contribution to antioxidant intake in the diet [43].

Pepino is a cultivated edible species, while *S. caripense* is occasionally harvested from the wild for its sweet fruits [5]. The extracts, even when not diluted, of both species did not affect the viability of macrophage cells, which is an indication of a lack of cytotoxicity [44]. The lack of cytotoxicity of *S. caripense* is in contrast to wild relatives of the genus *Solanum*, which are cytotoxic due to their high contents of glycoalkaloids and other antinutritional compounds [45,46], and therefore facilitates its use in breeding of the cultivated pepino [5]. Pepino and *S. caripense* raw extracts inhibited significantly the production of NO in macrophages stimulated by LPS, suggesting that the extracts modulate the production of NO formation and therefore may have an *in vivo* anti-inflammatory effect [47]. In this respect, chlorogenic acid is known to inhibit the inducible nitric oxide synthase [36].

The results obtained have failed to identify a single accession with high values for the traits studied, revealing that in our materials hydroxycinnamic acid content, reducing activity and NO inhibition in macrophage cells are not strongly related. This is probably a consequence that the different traits studied measure different aspects of the fruit quality of the samples. It is known that hydroxycinnamic acids contribute to antioxidant activity [48], but other antioxidant compounds present in the pepino flesh, like vitamin C or carotenoids [12,19], may also contribute significantly to the antioxidant activity. At the same time, the different antioxidant measures, due to the different nature of the chemical reactions involved, may produce considerable differences in the results [40]. Also, inhibition of NO production in LPS-stimulated macrophages does not exclusively depend on phenolics or antioxidant activity, as other bioactive compounds may be involved [49]. Therefore, if a pepino variety with high values for the different types of traits observed (phenolics content) is desired, we suggest either the screening of large collections of materials, or the intercrossing of complementary materials, like Puzol (with high ranks for hydroxycinnamic acids and inhibition of NO production in macrophages) and *S. caripense* E-7 (with high antioxidant activity), in order to perform selection in subsequent segregating generations [5,11].

## 4. Materials and Methods

### 4.1. Plant Material

**Figure 4:**
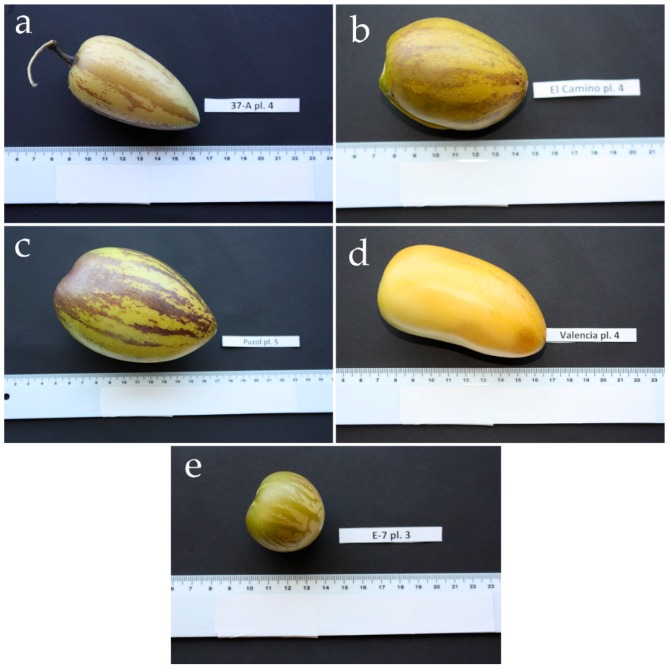
Fruit samples of the pepino (*S. muricatum*) and *S. caripense* accessions used. Pepino varieties correspond to 37-A (**a**); El Camino (**b**); Puzol (**c**); and Valencia (**d**); while *S. caripense* accession is E-7 (**e**). Scale is in cm. The plant (pl.) replicate from which samples were obtained is indicated.

Four accessions of pepino and one of *S. caripense* previously characterized at the morphological and molecular levels [2] were used for the present study. Pepino accessions were selected as representative of the diversity of pepino, while the *S. caripense* accession was included as representative of a wild relative of interest for pepino breeding [5] Pepino accession 37-A is a primitive local variety from Ecuador [2], El Camino is a cultivar developed in New Zealand [50], and Puzol and Valencia are two hybrid varieties used for salads or as a fresh fruit, respectively, both developed in Spain [51,52]. Finally, E-7 is an Ecuadorian accession of the wild pepino relative *S. caripense* [2]. The main characteristics of these accessions can be consulted in Table 5 and a picture of them is shown in Figure 4. Five clonal replicates of each accession were transplanted to a glasshouse in Valencia (Spain; global positioning system (GPS) coordinates: latitude 39°29′01″ N, longitude 0°20′27″ W) in January 2014 and were cultivated using the standard techniques for pepino cultivation in Mediterranean climates [53]. Manual pollination was performed on self-incompatible *S. caripense* plants [54] using pollen from another *S. caripense* accession in order to obtain fruit set. Further details on growing conditions can be consulted elsewhere [2].

### 4.2. Sample Preparation

Five fruit samples, each of which consisted of at least three fruits from one of the five clonal plants of each accession, were used for the analyses. Fruits were harvested when ripe (evaluated by the fruit size, color, and fruit skin glossiness) and brought to the laboratory, where they were washed, peeled, cut in slices, frozen in liquid N_2_, and stored at −80 °C until lyophilized. Powdered lyophilized tissue of the different fruits harvested of each plant was bulked and thoroughly mixed to form a sample.

### 4.3. Phenolic Composition

Subsamples of the lyophilized powdered fruit tissue (100 mg) were extracted with 1.5 mL of methanol:water:formic acid (70:29:1, *v*:*v*:*v*). The extracted phenolic samples were vortexed and subsequently sonicated in an ultrasonic bath for 60 min. The samples were stored overnight at 4 °C, after which they were sonicated again for 60 min and centrifuged at 10,000 rpm for 10 min in order to separate the supernatant from the solid residue. The supernatant was filtered through a 22 μm polyvinylidene (PVDF) filter before HPLC-DAD-MS^n^/ESI analysis.

Chromatographic analyses were carried out on a Kinetex column (5 µm, C18, 100 A, 150 mm × 4.6 mm; Phenomenex, Macclesfield, UK). Two solvents were used for the mobile phase: 1% acetic acid in water (A) and acetonitrile (B), starting with 1% B followed by 15% B in 15 min, 30% B at 30 min, maintained in 30% B at 40 min, changing to 95% B at 45 min, maintained in 95% B at 50 min, and decreasing to the initial conditions of 1% B at 55 min and 1% B at 60 min. The flow rate was 800 μL min^−1^, and the injection volume 5 μL. Spectral data from all peaks were obtained in the range 200–400 nm and chromatograms were recorded at 320 and 360 nm. The HPLC-DAD-MS^n^/ESI analyses were carried out as in Sánchez-Rodríguez *et al.* [24].

Identification of the peaks was done by analyzing both the UV-vis spectra, as well as the extracted-ion chromatograms of the ion current at *m/z* values corresponding to the [M − H]^−^ ions of the individual investigated compounds, as well as their fragmentation. The identified analytes were quantified by HPLC-DAD detection using the external standard method with calibration graphs, as a function of peak area-based concentration, detected at 320 nm for hydroxycinnamic acids and 360 nm for flavonoids, which are the wavelengths corresponding, respectively, to their maximum absorbance.

### 4.4. Antioxidant Activity

Antioxidant activity was measured using three different methods: 2,2-diphenyl-1-picrylhydrazyl hydrate (DPPH) free radical scavenging capacity, oxygen radical absorbance capacity (ORAC), adapted to microscale according to Mena *et al.* [55], and total reducing capacity (TRC) using the Folin–Ciocalteu reagent.

The DPPH method is based on the antioxidant capacity of the sample to neutralize the free radical DPPH in a lipophilic medium. The method determines the changes in absorbance at 515 nm after 50 min or reaction of the sample with DPPH radical. The assay was performed with 96-well microplates (Nunc) in an Infinite M200 Tecan microplate reader. The reaction starts by adding 2 μL of the diluted sample to the microplate well containing the stock solution (250 μL).

ORAC assay was performed according to Ou *et al.* [56]. The method consists of the measurement of fluorescence decay of the protein fluorescein due to its oxidation by peroxyl (ROO·) radicals formed by decomposition of the azo initiator 2,2’-azobis(2-amidinopropane) dihydrochloride (AAPH). In the presence of an antioxidant the decrease of fluorescence of the protein is diminished or inhibited, and that can be quantified by the Area under the Curve (AUC) of fluorescence time obtained in the 2 h of the reaction.

Standard curves of the antioxidant Trolox were used to express both ORAC and DPPH results, as mM Trolox/100 g dry weight.

Total reducing capacity (TRC) was determined using the Folin–Ciocalteu procedure as indicated in Plazas *et al.* [15]. Basically, 125 mg of lyophilized tissue were extracted with 15 mL of acetone:water:glacial acetic acid (70:29.5:0.5, *v*:*v*:*v*) at room temperature over a period of 24 h. In order to improve extraction the mixture was stirred continuously at room temperature. Subsequently the extracted samples were centrifuged at 3500 rpm for 5 min. An aliquot of 1.5 mL of the supernatant was subjected to a new round of centrifugation, this time at 10,000 rpm for 5 min. A new aliquot of 65 μL was subsequently mixed with 0.5 mL of water:Folin–Ciocalteu reagent (Sigma-Aldrich Chemie, Steinheim, Germany; 90:10, *v*:*v*) and, after 5 min, 0.5 mL of a sodium carbonate solution (60 g/L) was added to the mixture. After 90 min of incubation, the absorbance was measured at 750 nm. TRC was expressed as caffeic acid equivalents (g/kg of dry weight).

### 4.5. Anti-Inflammatory Activity

The anti-inflammatory activity was evaluated using the procedure described in Plazas *et al.* [15]. Subsamples of 500 mg of lyophilized fruit were extracted in 4 mL of methanol. In order to improve extraction, the mixture was placed in an ultrasonic bath for 30 min. Subsequently, the extracted samples were centrifuged at 2000 rpm for 5 min. An aliquot of the supernatant was sterilized by passing it through 0.2-µm sterile PTFE filters. Extract dilutions of 1:10 in sterile phosphate buffered saline (PBS) were prepared for each of the samples.

The murine macrophage cell line RAW 264.7 (European Collection of Authenticated Cell Cultures, Salisbury, UK) was used for the anti-inflammatory *in vitro* activity experiments [15]. Firstly, the effects on cell viability were tested. To do this, the effect of different extract dilutions on cell viability was evaluated using the MTT (3-[4,5-dimethylthiazol-2-yl]-2,5-diphenyl-tetrazolium bromide) assay. The RAW 264.7 macrophages were exposed to the raw extract, 1:1 or 1:10 dilutions in 96-well microplates for 24 h. After that, 20 µL of a 5 mg/mL solution of MTT were added to each well. Macrophage cells were incubated at 37 °C until formazan deposits (blue-colored) were observed. Formazan was dissolved in dimethyl sulfoxide (DMSO) and incubated for a few minutes at room temperature in an orbital shaker. Absorbance was measured at 490 nm, with decreases in absolute absorbance values being a measure of a reduction in cell viability.

The anti-inflammatory activity of the extracts was evaluated through the inhibition of the production of the free radical NO in stimulated murine macrophages [15]. The RAW 264.7 macrophages were cultured with raw extract, 1:1 or 1:10 dilutions in 96-well microplates for 1 h. Subsequently, lipopolysaccharide (LPS) (Sigma-Aldrich Chemie) at a concentration of 1 μg/mL was added in order to stimulate the macrophage cells. After a period of 24 h of incubation, a 100 μL aliquot of the culture medium was mixed with Griess reagent (Sigma-Aldrich Chemie), which gives a red color when NO is present [57]. The absorbance was read at 540 nm and the values of the extracts were compared to the control with no extract, which was assigned a relative value of 100%.

### 4.6. Data Analysis

A Euclidean distance matrix based on absence (0)/presence (1) of the phenolic compounds detected by HPLC-DAD-MS^n^/ESI was used for clustering analysis with the hierarchical clustering UPGMA (unweighted pair group method with arithmetic mean) method [58]. Euclidean distances calculation and cluster analysis were performed with NTSYS-pc 2.02i software [59] and the dendrogram was constructed using Treeview 1.6.6 [60]. Average values and standard errors were calculated for each accession for the quantitative data obtained. For viability and NO inhibition tests, the significance of the differences compared to the control were evaluated with a Dunnett’s *t*-test [61]. Varieties were ranked for their total contents in phenolics, antioxidant, and biological activity traits.

## 5. Conclusions

The fruit of the pepino and its wild relative *S. caripense* present significant quantities of phenolic acid derivatives, as well as remarkable antioxidant and biological activity, which might result in a beneficial effect on human health. The phenolic fraction of the flesh of the pepino and its wild relative *S. caripense* is mostly constituted by hydroxycinnamic acid derivatives, although modern pepino varieties have a different and richer profile of phenolic compounds than the wild *S. caripense* and the primitive pepino variety studied. Different accessions were ranked first for hydroxycinnamic acid content (modern pepino variety Valencia), antioxidant activity measures (*S. caripense* E-7), and biological activity (primitive pepino variety 37-A). This suggests that selection of larger collections or the development of breeding programs will have to be undertaken if varieties with high values are desired for the three traits measured here. Our results provide relevant information about the phenolics composition, antioxidant, and biological activities of a diverse set of pepino cultivars as well as its wild relative *S. caripense*. This information will contribute to the enhancement of this neglected crop.

## Figures and Tables

**Table 1:** Rt, MS: [M − H]^−^, MS^2^ [M − H]^−^ and relative abundance (%; calculated based on the intensity of the main base peak in the MS^2^ fragmentation pattern, which is considered as 100%) of phenolic compounds identified (marked by an X) in fruit samples of pepino (*S. muricatum*) and its wild relative *S. caripense* samples.

Peak	Compound	Rt	[M − H]^−^	MS^2^[M − H]^−^, *m/z* (%)	37-A	El Camino	Puzol	Valencia	E-7
**1**	Di-caffeoyl-quinic acid I	15.2	515	353 (54), 191(100)	–	–	–	–	X
**2**	3-Caffeoyl-quinic acid	16.4	353	191 (100), 179 (42)	X	X	X	X	X
**3**	Caffeoyl-hexoside I	17.4	341	179 (100), 135 (21)	–	X	X	X	X
**4**	Caffeoyl-hexoside II	17.6	341	179 (64), 135 (9)	–	X	X	X	–
**5**	Di-caffeoyl-quinic acid II	18.4	515	353 (78), 191 (88)	–	–	–	–	X
**6**	Caffeoyl-hexoside III	18.4	341	179 (100), 135 (19)	–	X	X	X	–
**7**	Feruloyl-hexoside	19.2	355	193 (100), 175 (53)	–	–	X	–	–
**8**	Caffeoyl-di-hexoside	19.3	503	341 (36), 179 (100)	–	X	X	X	–
**9**	Caffeoyl-hexose IV	19.8	341	179 (100), 135 (12)	X	X	X	X	X
**10**	5-Caffeoyl-quinic acid	20.6	353	191 (100)	X	X	X	X	X
**11**	4-Caffeoyl-quinic acid	20.7	353	173 (100)	X	X	X	X	X
**12**	*p*-Coumaroyl-di-hexoside	22.0	487	325 (14), 163 (100)	–	X	X	X	X
**13**	Feruloyl-hexoside	22.3	355	193 (100), 175 (56)	–	X	X	X	–
**14**	Caffeoyl-quinic acid isomer	23.0	353	191 (100)	–	X	–	–	–
**15**	Feruloyl-dihexoside	23.2	517	235 (50), 193 (100), 175 (76)	–	X	X	X	–
**16**	Sinapoyl-di-hexoside	23.4	547	265 (82), 324 (43), 223 (28)	–	X	–	–	–
**17**	Feruloyl-hexoside	24.4	355	193 (100), 175 (19)	–	–	–	X	–
**18**	Caffeoyl-hexoside derivative	27.8	441	341 (100)	–	–	–	X	–
**19**	Di-caffeoyl-quinic acid	30.6	515	353 (100), 191 (12)	–	–	X	–	–
**20**	Sinapoyl-quinic acid derivative	31.1	577	415 (100), 353 (13), 191 (9)	–	X	–	–	–
**21**	Feruloyl-di-hexoside derivative	31.7	517	323 (100), 193 (41), 179 (19)	–	–	X	–	–
**22**	Di-caffeoyl-quinic acid	32.9	515	353 (100), 191 (2)	–	X	X	X	–
**23**	Caffeoyl-hexoside derivative	34.6	423	179 (100), 135 (28)	–	–	–	X	–
**24**	Caffeoyl-sinapoyl-quinic acid	35.7	559	397 (100), 223 (18), 173 (3)	X	X	X	X	X

**Table 2:** Compounds quantified (mg/g dry weight) in fruit samples of pepino (*S. muricatum*) and its wild relative *S. caripense* samples by HPLC-DAD.

Peak	Compound	*S. muricatum*				*S. caripense*
		37-A	El Camino	Puzol	Valencia	E-7
2	3-Caffeoyl-quinic acid	0.90 ± 0.31	<LOQ	<LOQ	<LOQ	<LOQ
9	Caffeoyl-hexose IV	0.07 ± 0.01	<LOQ	<LOQ	<LOQ	<LOQ
10	5-Caffeoyl-quinic acid	<LOQ	0.89 ± 0.46	1.44 ± 0.24	1.38 ± 0.35	0.19 ± 0.03
11	4-Caffeoyl-quinic acid	0.03 ± 0.01	<LOQ	<LOQ	<LOQ	<LOQ
12	*p*-Coumaroyl-di-hexose	n.d.	0.06 ± 0.01	0.14 ± 0.04	0.41 ± 0.05	0.06 ± 0.01
13	Feruloyl-hexose	n.d.	0.14 ± 0.04	0.28 ± 0.08	<LOQ	n.d.
14	5-Caffeoyl-quinic acid isomer	n.d.	0.03 ± 0.01	n.d.	n.d.	n.d.
15	Feruloyl-dihexose	n.d.	0.26 ± 0.04	0.16 ± 0.02	0.37 ± 0.07	n.d.
16	Sinapoyl-di-hexose	n.d.	0.05 ± 0.01	n.d.	n.d.	n.d.
18	Caffeoyl-hexose derivative	n.d.	n.d.	n.d.	0.06 ± 0.01	n.d.
19	Di-caffeoyl-quinic acid	n.d.	<LOQ	0.06 ± 0.01	<LOQ	n.d.
20	Sinapoyl-quinic acid derivative	n.d.	0.05 ± 0.02	n.d.	n.d.	n.d.
24	Caffeoyl-sinapoyl-quinic acid	0.10 ± 0.02	0.04 ± 0.01	0.09 ± 0.01	0.13 ± 0.02	1.13 ± 0.15
–	Total hydroxycinnamic acids	1.11 ± 0.08	1.51 ± 0.07	2.17 ± 0.07	2.35 ± 0.10	1.37 ± 0.06

n.d.: not detected; <LOQ: detected but present at concentrations lower than the limit of quantification (LOQ). Values are expressed as mean ± standard error (SE) of five independent samples for each variety.

**Table 3:** Antioxidant activity of fruit samples of pepino (*S. muricatum*) and its wild relative *S. caripense* samples (*n* = 5) using the 2,2-diphenyl-1-picrylhydrazyl hydrate (DPPH) free radical scavenging capacity, oxygen radical absorbance capacity (ORAC), and total reducing capacity (TRC) based on the Folin–Ciocalteu reagent methods.

Accession	ORAC (µmol Trolox/g d.w.)	DPPH (µmol Trolox/g d.w.)	TRC (µmol caffeic acid/g d.w.)
*S. muricatum*			
37-A	83.5 ± 7.0	26.1 ± 3.4	99.2 ± 11.7
El Camino	76.0 ± 2.6	22.2 ± 1.5	73.6 ± 1.7
Puzol	80.9 ± 8.2	34.5 ± 2.1	66.2 ± 4.8
Valencia	51.9 ± 11.9	29.2 ± 2.1	76.8 ± 6.7
*S. caripense*			
E-7	170.7 ± 22.9	36.3 ± 2.9	127.9 ± 4.8

**Table 4:** Ranking (ordered from highest to lowest) for total content of hydroxycinnamic acids, antioxidant activity measures (ORAC, DPPH, and TRC), and inhibition of NO production in the raw extracts (1:1) of lyophilized samples of the different accessions studied of pepino (*S. muricatum*) and its wild relative *S. caripense*, and sum of ranks for each accession.

Accession	Hydroxycinnamic Acids	ORAC	DPPH	TRC	NO Inhibition
*S. muricatum*					
37-A	5	2	4	2	1
El Camino	3	4	5	4	5
Puzol	2	3	2	5	2
Valencia	1	5	3	3	4
*S. caripense*					
E-7	4	1	1	1	3

**Table 5:** Pepino (*S. muricatum*) and its wild relative *S. caripense* accessions used in the present study and main fruit characteristics.

Accession	Origin	Main Use	Fruit Shape	Fruit Weight (g)	Soluble Solids Content (%)
*S. muricatum*					
37-A	Ecuador	Fresh fruit	Conical	72 ± 9	5.4 ± 0.5
El Camino	New Zealand	Fresh fruit	Heart-shaped	127 ± 12	6.7 ± 0.5
Puzol	Spain	Salads	Ellipsoid	213 ± 24	7.2 ± 0.4
Valencia	Spain	Fresh fruit	Elongated	192 ± 22	7.6 ± 0.6
*S. caripense*					
E-7	Ecuador	Occasionally picked for its sweet fruits	Round	19 ± 2	10.1 ± 0.9

## Abbreviations

The following abbreviations are used in this manuscript:

ORAC	Oxygen radical absorbance capacity
DPPH	2,2-Diphenyl-1-picrylhydrazyl hydrate
TRC	Total reducing capacity
AUC	Area under the curve
MTT	3-[4,5-Dimethylthiazol-2-yl]-2,5-diphenyl-tetrazolium bromide
